# LIMD1 is induced by and required for LMP1 signaling, and protects EBV-transformed cells from DNA damage-induced cell death

**DOI:** 10.18632/oncotarget.23676

**Published:** 2017-12-26

**Authors:** Ling Wang, Mary E.A. Howell, Brooke McPeak, Katrina Riggs, Carissa Kohne, Jether Uel Yohanon, Daniel E. Foxler, Tyson V. Sharp, Jonathan P. Moorman, Zhi Q. Yao, Shunbin Ning

**Affiliations:** ^1^ Center of Excellence for Inflammation, Infectious Diseases and Immunity, Quillen College of Medicine, East Tennessee State University, Johnson City 37614, TN, USA; ^2^ Department of Internal Medicine, Quillen College of Medicine, East Tennessee State University, Johnson City 37614, TN, USA; ^3^ Centre for Molecular Oncology, Barts Cancer Institute, University of London, London EC1M 6BQ, UK; ^4^ Hepatitis (HCV/HIV) Program, James H Quillen VA Medical Center, Johnson City 37614, TN, USA

**Keywords:** LIMD1, p62, DDR, autophagy, EBV

## Abstract

LIMD1 (LIM domain-containing protein 1) is considered as a tumor suppressor, being deregulated in many cancers to include hematological malignancies; however, very little is known about the underlying mechanisms of its deregulation and its roles in carcinogenesis. Epstein-Barr Virus (EBV) is associated with a panel of malignancies of lymphocytic and epithelial origin. Using high throughput expression profiling, we have previously identified LIMD1 as a common marker associated with the oncogenic transcription factor IRF4 in EBV-related lymphomas and other hematological malignancies. In this study, we have identified potential conserved IRF4- and NFκB-binding motifs in the LIMD1 gene promoter, and both are demonstrated functional by promoter-reporter assays. We further show that LIMD1 is partially upregulated by EBV latent membrane protein 1 (LMP1) via IRF4 and NFκB in EBV latency. As to its role in the setting of EBV latent infection, we show that LIMD1 interacts with TRAF6, a crucial mediator of LMP1 signal transduction. Importantly, LIMD1 depletion impairs LMP1 signaling and functions, potentiates ionomycin-induced DNA damage and apoptosis, and inhibits p62-mediated selective autophagy. Taken together, these results show that LIMD1 is upregulated in EBV latency and plays an oncogenic role rather than that of a tumor suppressor. Our findings have identified LIMD1 as a novel player in EBV latency and oncogenesis, and open a novel research avenue, in which LIMD1 and p62 play crucial roles in linking DNA damage response (DDR), apoptosis, and autophagy and their potential interplay during viral oncogenesis.

## INTRODUCTION

Epstein-Barr Virus (EBV) infection is associated with more than 50% of AIDS-related lymphomas (ARLs) and other malignancies such as nasopharyngeal carcinoma, up to 400 thousand cases each year as estimated by the World Health Organization (WHO) [1]. The EBV Latent membrane protein 1 (LMP1) is a pleiotropic factor that promotes cell growth and transformation *in vitro* as well as in transgenic mice [2]. LMP1 oncogenicity is attributed by its ability to activate multiple oncogenic transcription factors, including NFκB that interacts with other EBV oncoproteins to form viral super-enhancers to regulate expression of a large scale of host genes involved in lymphoblastoid B-cell growth and survival [3].

The LIM domain-containing protein 1 (LIMD1) is a member of the ZYXIN family [4]. Like the oncogenic transcription factor interferon regulatory factor 4 (IRF4), overexpression of LIMD1 is a hallmark of ABC subtype of diffuse large B cell lymphoma (DLBCL) [5]. LIMD1 is involved in the assembly of numerous protein complexes by acting as an adaptor protein that interacts with various proteins such as Rb [6], TRAF6 [7], p62/SQSTM1 [8], VHL and PHD [9, 10], and LATS and WW45 [11], and participates in myriad cellular processes including cell fate determination, cytoskeletal organization, osteoclastogenesis [8], repression of gene transcription, cell-cell adhesion, cell differentiation, proliferation and migration. Interaction of LIMD1 with TRAF6 enhances the ability of TRAF6 to activate AP1 and negatively regulates the canonical Wnt receptor signaling pathway in osteoblasts [7], and interaction with p65 negatively regulates NFκB activity in human non-small cell lung cancer cells [12]. Our previous study has shown that LIMD1 and IRF4 expression levels positively correlate in different hematological malignancies, including EBV-associated lymphomas [13]. However, the mechanisms underlying its regulation and its role in the setting of EBV infection remain uninvestigated.

DNA damage is directly linked to a large range of human diseases, including aging and cancer [14–16], and usually has severe effects on the cell—triggering cell-cycle arrest, cell death or tumorigenesis. Reactive oxygen species (ROS), which can be produced by diverse conditions of stress such as chronic viral infection and cancer hypoxia [17, 18], are one of the major causes of DNA damage [19]. Most cancers, if not all, harbor deficient DNA repair mechanisms, resulting in increased genomic instability and less capacity to respond to DNA damages; therefore they heavily rely on alternative DNA repair mechanisms for survival [14]. Deficiency in DNA repair mechanisms also results in resistance to conventional chemotherapeutic agents in cancer cells [20, 21], in which DNA damage-induced autophagy plays a cryoprotective role [22, 23].

An increasing body of evidence indicates that autophagy and DNA damage closely crosstalk, in which the selective autophagy adaptor p62 (known as SQSTM1/Sequestosome-1) plays a key role [24–27]. As a part of the DNA damage response (DDR), autophagy promotes DNA damage repair by targeting DDR-related proteins including p62 for degradation, contributing to the maintenance of genomic stability in aging and cancer [22, 27]. Many cancer cells have high apoptotic thresholds, so autophagy serves as a survival mechanism that allows these cancer cells to escape apoptotic or necrotic death in response to metabolic crisis. Thus, the heavy reliance of many cancer cells on autophagy for survival suggests inhibiting autophagy in these cells may be a promising therapeutic target [23].

In this study, we show evidence that LIMD1 is upregulated by LMP1 via NFκB and IRF4 axes in EBV latency. We further show that LIMD1 is required for LMP1 signal transduction and function. More importantly, LIMD1 depletion potentiates ionomycin-induced DNA damage, and impairs p62-mediated selective autophagy.

## RESULTS

### IRF4, NFκB, and LMP1 transactivate the LIMD1 gene promoter

**Figure 1:**
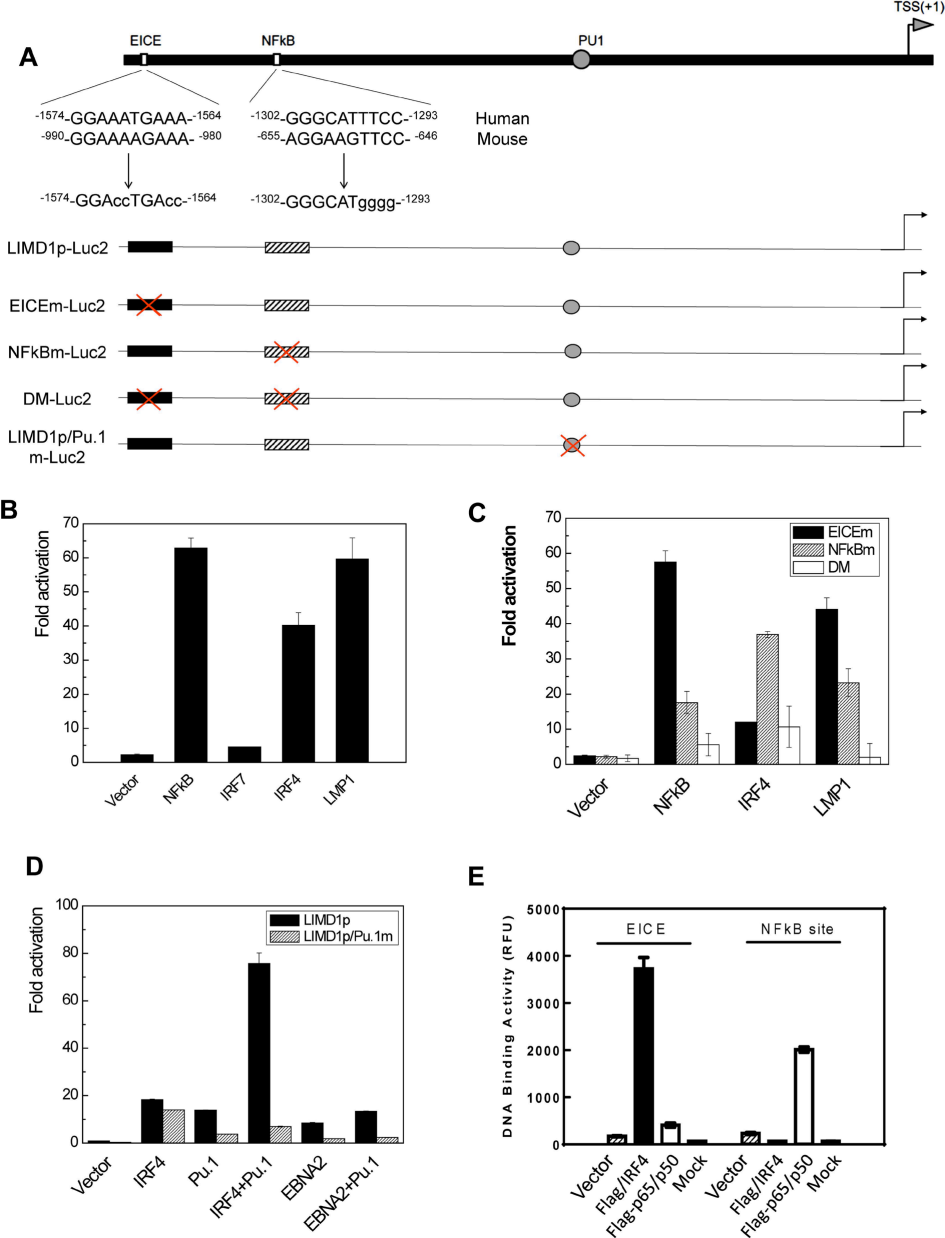
LMP1, NFκB and IRF4 transactivates LIMD1 gene promoter (**A**) A diagram showing the LIMD1 promoter construct pGL4.10/LIMD1p(-1990/+50)-Luc2 and its mutants. (**B**) LMP1, NFκB and IRF4 transactivate the wild type pGL4.10/LIMD1p(-1990/+50)-Luc2. (**C**) The putative NFκB- and IRF4-binding sites in the LIMD1 promoter are functional. 293 cells in 24-well plates were transfected with 150 ng IRF4, 150 ng p65 plus p50, or 10 ng LMP1, 40 ng pGL4.10/LIMD1p(-1990/+50)-Luc2 or its mutant with the Pu.1-binding site mutated, and 10 ng Renilla. Dual luciferase assay was performed. The ability of the vector control to activate the promoter construct was set to 1. (**D**) Pu.1 and IRF4 synergically transactivate the LIMD1 promoter. 293 cells in 24-well plates were transfected with 150 ng IRF4, 150 ng EBNA2, 150 ng Pu.1 or their combinations, 40 ng pGL4.10/LIMD1p(-1990/+50)-Luc2 or it mutant with the Pu.1-binding site mutated, and 10 ng Renilla. Dual luciferase assays and data processing were performed as above. (**E**) 293 cells in 100-mm dishes were transfected with Flag-IRF4, Flag-p65+Flag-p50, or vector control, or mock transfected. Cells were then subjected to immunoprecipitation with anti-Flag M2 after 48 h, followed by ChIP assays. For each sample, DNA pellets were dissolved in 200 µl ddH_2_O, and 15 µl was used for qPCR using the primers for LIMD1 promoter EICE and NFκB-binding site. DNA-binding activity is represented by relative fluorescence units (RFU). Results are the averages ± standard error (SE) of duplicates. Representative results from at least three independent experiments are shown.

We have previously shown that LIMD1 expression correlates with IRF4 in hematological malignancies [13], suggesting that LIMD1 may be transcriptionally regulated by IRF4. One of the IRF4 consensus binding site is ETS/ISRE-consensus element (EICE), which has the consensus sequence 5-GGAANNGAAA-3 fusing the ETS-binding motif (5-GGAA-3) with the IRF4-binding motif (5-AANNGAAA-3) [28]. To investigate this possibility that IRF4 regulates LIMD1 transcription, we analyzed its promoter region, and identified potential EICE and NFκB-binding sites (Figure 1A), in addition to the known Pu.1-binding site [29]. Promoter-reporter assay results further showed that IRF4, NFκB or LMP1 alone can activate the human LIMD1 promoter, but IRF7 had no significant effect on it (Figure 1B). To confirm the two potential sites are functional, we created a panel of point mutants in these sites that were then subjected to promoter-reporter assays (Figure 1A). The results show that mutation of either of these two sites impairs the LIMD1 promoter activity, and mutation of both disables LMP1-stimulated promoter activity (Figure 1C).

We also evaluated the potential cooperation between IRF4 and Pu.1 in transactivating the LIMD1 promoter, with the LIMD1 promoter construct mutated in the Pu.1-binding site [29]. As shown in Figure 1D, cotransfection of IRF4 with Pu.1 results in dramatically increased activity than IRF4 or Pu.1 alone. EBNA2, an EBV nuclear antigen that is another Pu.1-binding partner [30], also transactivate the LIMD1 promoter but not the mutant with Pu.1-binding site mutated; however, no synergic effect between EBNA2 and Pu.1 was detected. These data indicate that IRF4 and Pu.1 can transactivate the LIMD1 promoter in a synergic manner.

We further performed ChIP assays to assess the binding of IRF4 and NFκB with the endogenous LIMD1 promoter in 293 cells. IRF4-DNA and NFκB-DNA complexes were pulled down with the Flag M2 antibody, and the recovered DNA fragments were subjected to real-time PCR amplification for a fragment containing the potential EICE and NFκB-binding sites. Results indicate that IRF4 and NFκB bind to the LIMD1 promoter in 293 cells (Figure 1E).

In conclusion, our results demonstrate that EBV LMP1 transactivates the LIMD1 promoter via IRF4 and NFκB signaling axes.

### LIMD1 expression is upregulated by IRF4 and NFκB downstream of LMP1 signaling

**Figure 2:**
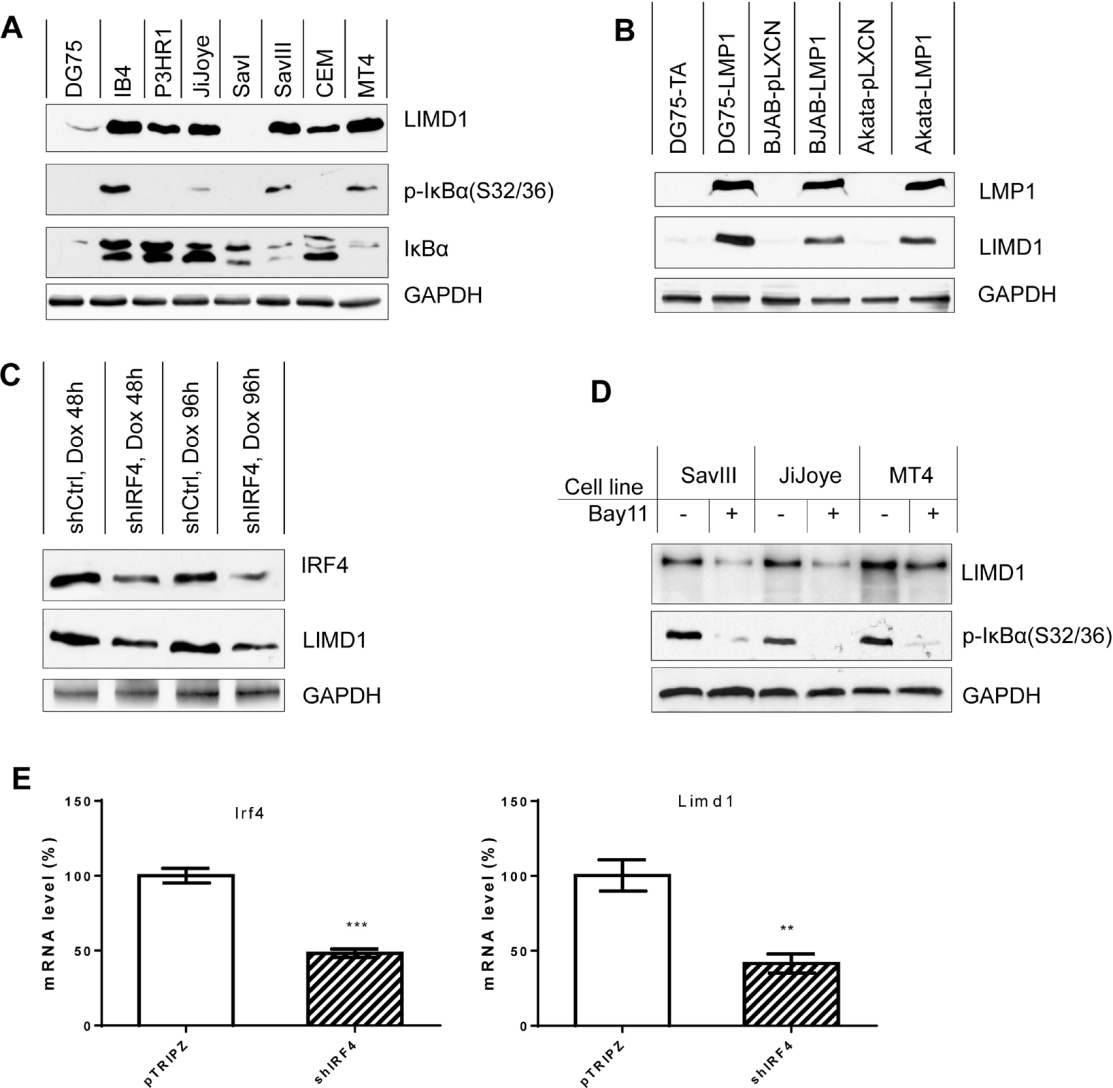
LIMD1 is upregulated by NFκB and IRF4 in virus-transformed cells (**A**) LIMD1 expression is correlated with NFκB activity in B and T cell lines. (**B**) LMP1 induces LIMD1 expression. BJAB and Akata stable cell lines expressing LMP1 or control were generated by transfecting with pLXCN/Flag-LMP1 expression and control plasmids and selected with 2 mg/ml G418 for two weeks, and then subjected to immunoblotting analysis for LIMD1 protein. LMP1 in DG75 stable cells was induced by 1 µg/ml doxycycline for 48 h before collection. All cells were treated with 5 µM MG132 for 6 h before collection. (**C**) Knockdown of IRF4 in IB4 cells decreases LIMD1 protein level in EBV-transformed cells. IB4 stable cell lines expressing pTRIPz/shIRF4 or control were induced with 1 µg/ml doxycycline, and IRF4 and LIMD1 protein levels were evaluated. (**D**) Inhibition of NFκB activity decreases LIMD1 protein levels in virus-transformed cells. NFκB activity was inhibited with the NFκB-specific inhibitor Bay11-7085 at the concentration of 2.5 µM for 48 h. (**E**) IRF4 depletion by IRF-specific shRNA downregulates LIMD1 mRNA expression in JiJoye cells. JiJoye cells stably expressing control and IRF4 shRNAs were treated with 1 µg/ml doxycycline to induce shRNA expression for 96 h. Total RNAs were extracted for qPCR to quantitate IRF4 and LIMD1 mRNA expression. The average mRNA levels of the duplicates in shControl-expressing cells were set to 100%.

We next evaluated the regulation of LIMD1 expression by IRF4, NFκB, and LMP1. We have previously shown that LIMD1 expression is associated with IRF4 in EBV-positive and negative B lymphoma cell lines [13]. We further show here that the LIMD1 protein level is associated with NFκB activity (as indicated by IκBα phosphorylation), in B and T lymphoma cell lines (Figure 2A). In EBV-negative B cells and type I latency where p-IκBα(S32/36) is not detected, LIMD1 levels are low or undetectable; however, in EBV type III latency and in MT4 T cell line where p-IκBα(S32/36) is considerable, LIMD1 expression is readily detectable (Figure 2A). Notably, in P3HR1 cells derived from the parental JiJoye cell line but lacking LMP1 expression due to the deletion of the EBNA2 gene, LIMD1 is also expressed at a considerable level; it is however consistently lower than that in the parental cell line JiJoye, indicating that mechanisms other than LMP1 signaling may contribute to the induction of LIMD1 expression in EBV latency.

We then assessed LIMD1 expression in several cell lines including DG75, BJAB and Akata that stably express LMP1 vs. vector control. The data show that stable expression of LMP1 elevates LIMD1 protein levels in these cell lines (Figure 2B). However, transient expression of high level of LMP1 promotes proteasome-mediated degradation of LIMD1 (see Figure 4). We then assessed the LIMD1 protein levels in IB4 cell line stably expressing IRF4 or scramble control short hairpin RNAs (shRNAs) that were induced by doxycycline (Dox). The shIRF4 cloned in pTRIPz and control were used in our previous publication [31]. Results show that IRF4 deficiency results in a consistent decrease in endogenous LIMD1 protein levels in IB4 cells (Figure 2C). We have further blocked endogenous NFκB activity in EBV-positive cell lines with type III latency and in HTLV1-positive MT4 cell line using the IKKβ-specific inhibitor Bay11-7085 and then evaluated LIMD1 protein levels. Results show that NFκB blockage inhibits LIMD1 expression (Figure 2D).

**Figure 3:**
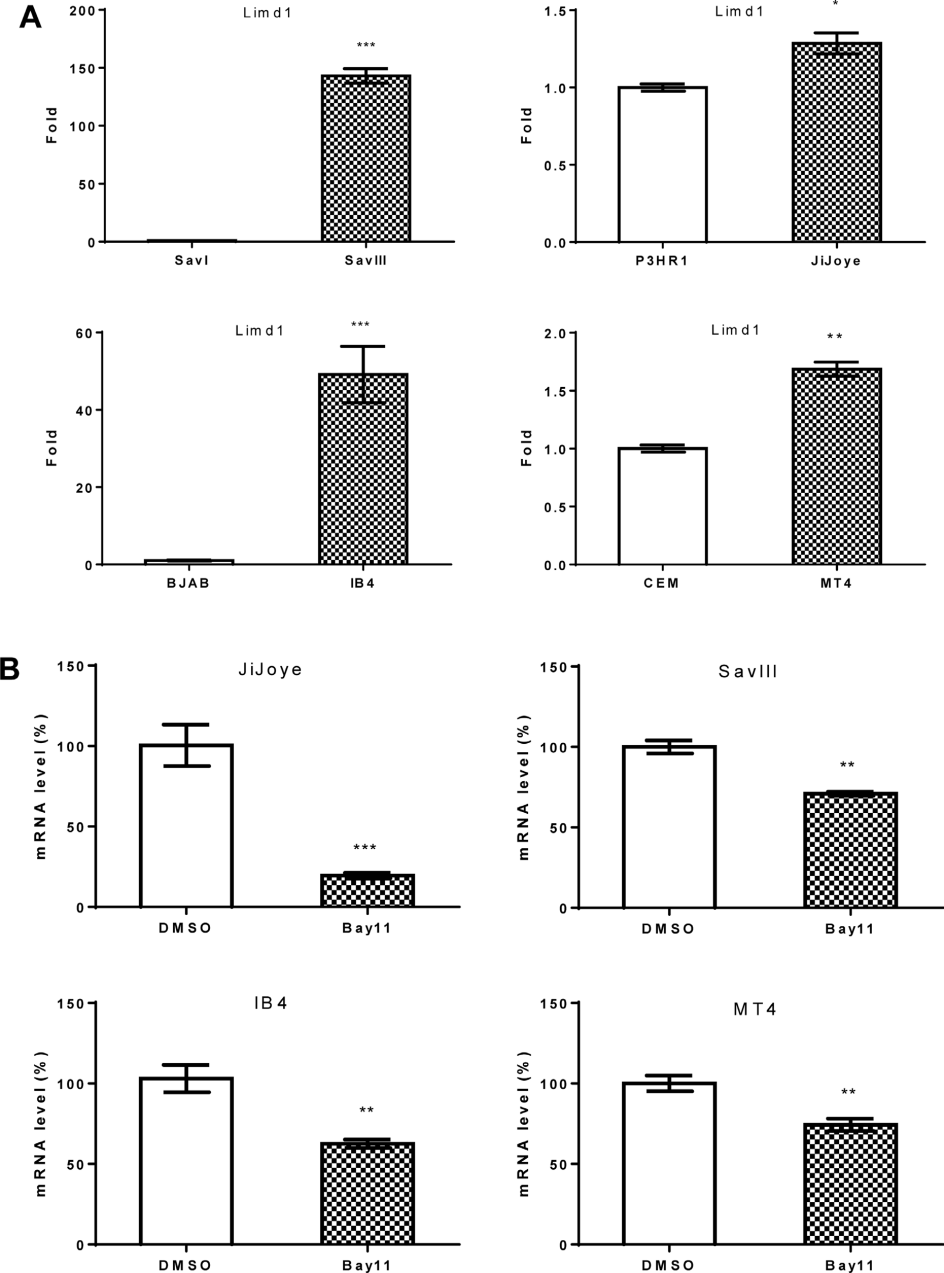
LIMD1 mRNA level is associated with EBV latency and upregulated by LMP1 (**A**) RNA was extracted from indicated different pair of cell lines, and LIMD1 mRNA expression level was evaluated by real-time qPCR. The average mRNA levels of the duplicates in SavI, BJAB, P3HR1, and CEM were set to 1. (**B**) Cell lines with high endogenous NFκB activity were treated with 2.5 µM Bay11-7085 for 48 h. RNA was then extracted for real-time qPCR analysis for LIMD1 expression. The average LIMD1 mRNA levels of the duplicates in DMSO-treated cells were set to 100%. The LIMD1 mRNA levels decreased by Bay11-7085 treatment are shown as percentage of those with corresponding DMSO controls. Statistical analysis was performed on results from three independent experiments.

**Figure 4:**
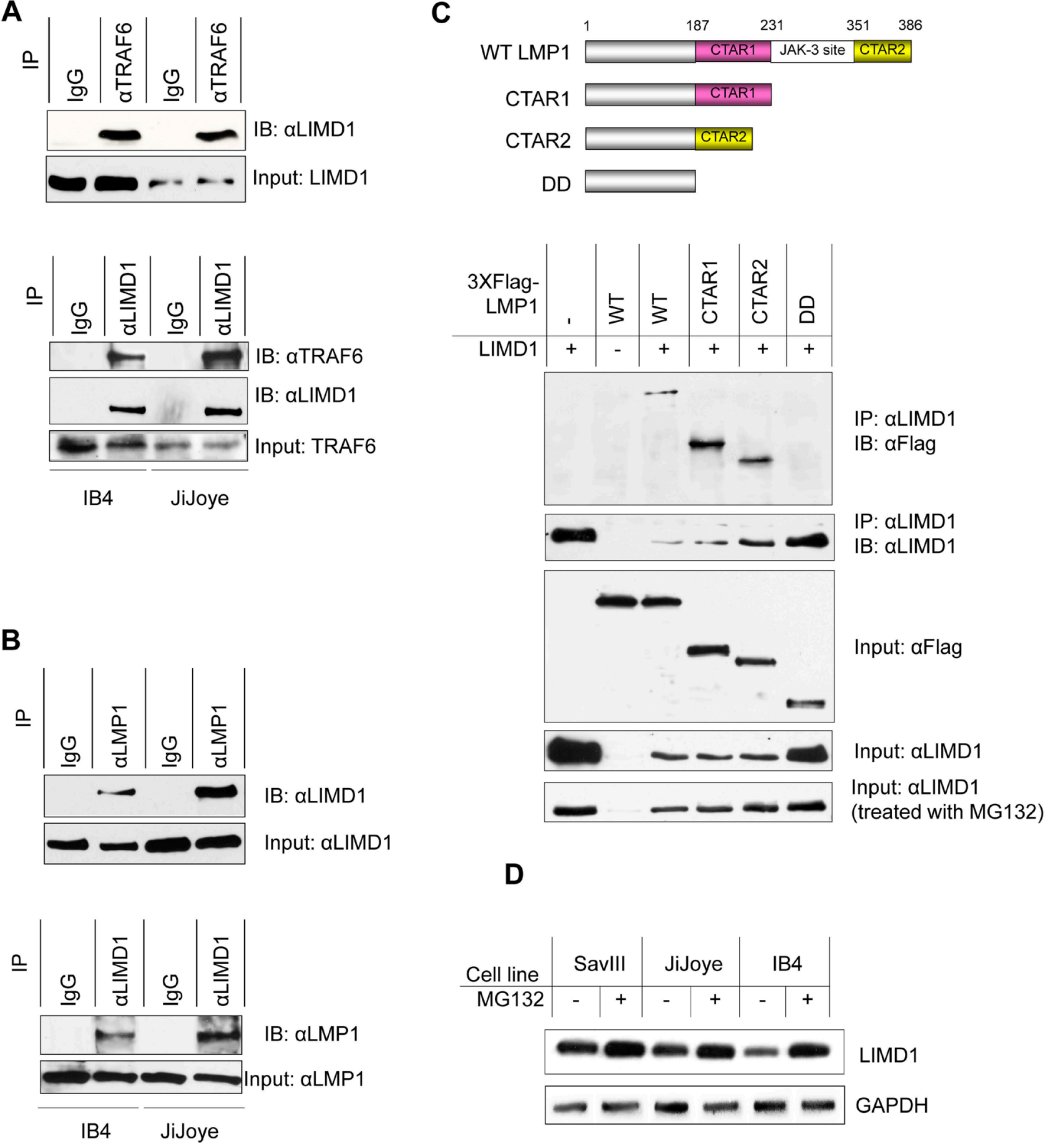
LIMD1 interacts with TRAF6 and LMP1 in EBV latency (**A**) IB4 and JiJoye cell lysates were subjected to immunoprecipitation with the TRAF6 antibody clone 1H4L2 and then immunoblotting with the LIMD1 antibody clone H-4 (upper), or vice versa (bottom). (**B**) IB4 and JiJoye cell lysates were subjected to immunoprecipitation with the LMP1 antibody clone CS1-4 and then immunoblotting with the LIMD1 antibody H-4 (upper), or vice versa (bottom). (**C**) Upper panel: A diagram of the LMP1 deletion mutants for immunoprecipitation. Lower panel: 3XFlag-LMP1 and its mutants were co-transfected with pcDNA3/LIMD1 into 293T cells. After 48 h, cells were collected and cell lysates were subjected to immunoprecipitation with the LIMD1 antibody clone H-4, and immunoprecipitants were probed with the Flag antibody M2. For MG132 treatment, MG132 was added at a final concentration of 10 µM for 6 h before collection. (**D**) Cells were treated with MG132 at a final concentration of 10 µM for 6 h before collection. Cell lysates were subjected for IB with the indicated antibodies.

We also evaluated LIMD1 regulation at the transcriptional level using real-time quantitative PCR, and the data indicate that LIMD1 mRNA and protein are consistent in their regulation by NFκB and IRF4 in these cell lines under normal culture conditions (Figure 2E; Figure 3).

Taken together, our results demonstrate that LIMD1 is upregulated by NFκB and IRF4 downstream of LMP1 signaling pathway.

### LIMD1 physically interacts with LMP1 and TRAF6

The upregulation of LIMD1 by LMP1 signaling implicates that LIMD1 may play a role in EBV latency and oncogenesis. As an adaptor protein, LIMD1 interacts with TRAF6, and is a positive regulator of NFκB and AP1 activation; however, it negatively regulates the canonical Wnt receptor signaling pathway in osteoclastogenesis [7, 8]. The closest family member, Ajuba, also positively regulates IL1-stimulated NFκB activation [32]. Thus, we first verifed that endogenous LIMD1 and TRAF6 clearly interact in EBV-transformed cells (Figure 4A). We have further shown that endogenous LIMD1 interacts with LMP1 as well (Figure 4B). To define the specificity of LIMD1/LMP1 interaction, we transiently expressed a panel of 3XFlag-LMP1 deletion mutants (Figure 4C, upper panel) with LIMD1 in 293T cells, and cell lysates were collected for immunoprecipitation. Our results show that both LMP1 CTAR1 and CTAR2 interact with LIMD1, and deletion of both ablated its ability to interact with LIMD1 (Figure 4C, lower panel).

Of note, our results consistently show that overexpression of the full length of LMP1, LMP1 CTAR1 or CTAR2 results in significantly lower levels of LIMD1 protein (Figure 4C, lower panel). This downregulation occurred at the post-translational level since it was prevented by treatment of the cells with MG132, a 26S proteosome specific inhibitor (Figure 4C, lower panel). Similar effects of MG132 on endogenous LIMD1 proteins were observed in EBV^+^ B cells with high levels of LMP1 (Figure 4D). These observations imply that high levels of LMP1 promote LIMD1 degradation through a proteosome-dependent pathway. We will further investigate these findings and the underlying mechanism, including identification of LIMD1 ubiquitination sites responsible for its stability regulation, in a separate project.

Together, these results demonstrate that LIMD1 interacts with both TRAF6 and LMP1 in EBV latency, and imply that high levels of LMP1 downregulate LIMD1 at the post-translational level.

### LIMD1 is required for LMP1 signal transduction and target gene regulation

**Figure 5:**
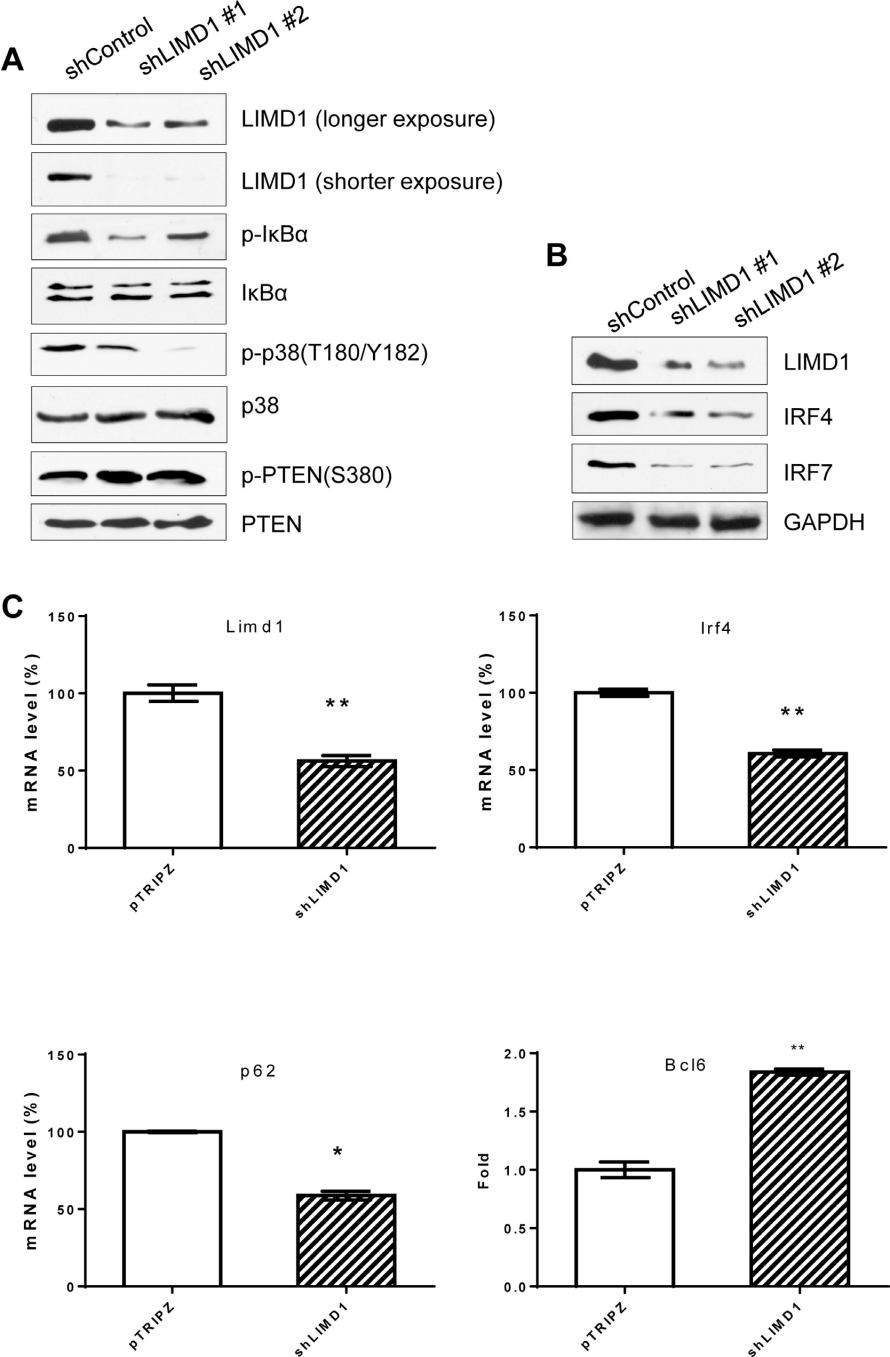
LIMD1 is required for LMP1 signal transduction and regulation of target genes (**A**) and (**B**) IB4 cell lines stably expressing LIMD1 shRNA #1 and #2 were induced by 1 µg/ml doxycycline for 48 h for LIMD1 shRNA expression, and cell lysates were then subjected to immunoblotting for analysis of the LMP1 downstream pathway activity and target gene expression. (**C**) LIMD1, IRF4, p62 and Bcl6 in IB4 stable cell line expressing LIMD1 shRNA #1 were also analyzed at mRNA levels using real-time quantitative PCR. The average mRNA levels of the duplicates in shControl-expressing cells were set to 100%.

Since LIMD1 interacts with TRAF6, a crucial mediator for LMP1 activation of NFκB and AP1, we next evaluated the requirement of LIMD1 for LMP1 signal transduction. To this end, we first depleted endogenous LIMD1 expression using LIMD1-specific shRNAs. As shown in Figure 5A, we achieved high knockdown efficiency using two out of six LIMD1 shRNA constructs. After selection of the cells with puromycin and induction of shRNA expression by Dox, we assessed NFκB and AP1 activity by immunoblotting for phosphorylation of IκBα and p38. Results show that depletion of LIMD1 significantly attenuates phosphorylation of both IκBα and p38 that requires TRAF6, but did not have detectable effects on PTEN and its phosphorylation at S380 downstream of LMP1/PI3K. These results indicate that LIMD1 is specifically required for LMP1/TRAF6-mediated signal transduction.

We next assessed the requirement of LIMD1 in regulation of LMP1 target gene expression. We performed immunoblotting and real-time qPCR for selected LMP1 targets, including IRF4, IRF7, and Bcl6 [33–35]. Our data show that protein levels of IRF4 and IRF7 are significantly downregulated in LIMD1 shRNA-expressing cells, compared with control shRNA-expressing cells (Figure 5B), and Bcl6 mRNA levels are increased (Figure 5C). We also show that p62 mRNA is downregulated in LIMD1-deficient cells (Figure 5C).

Taken together, these results indicate that LIMD1 is required for LMP1 signal transduction and regulation of its target genes.

### LIMD1 depletion potentiates DNA damage-induced cell death and inhibits autophagy

EBV latent infection causes genomic instability through different mechanisms independently mediated by EBNA1 and EBNA3C, and LMP1 [36]; among these LMP1 inhibits DNA repair in epithelial cells through distinct mechanisms, including its ability to inhibit DNA-PK/AMPK signaling [37], to inhibit PI3K/Akt/FOXO3a signaling [38], and to downregulate expression of ATM. ATM is a key PI3K-like kinase that phosphorylates multiple factors, such as CHK2, 53BP1, BRCA1 and H2AX, for double-stranded DNA repair [39].

**Figure 6:**
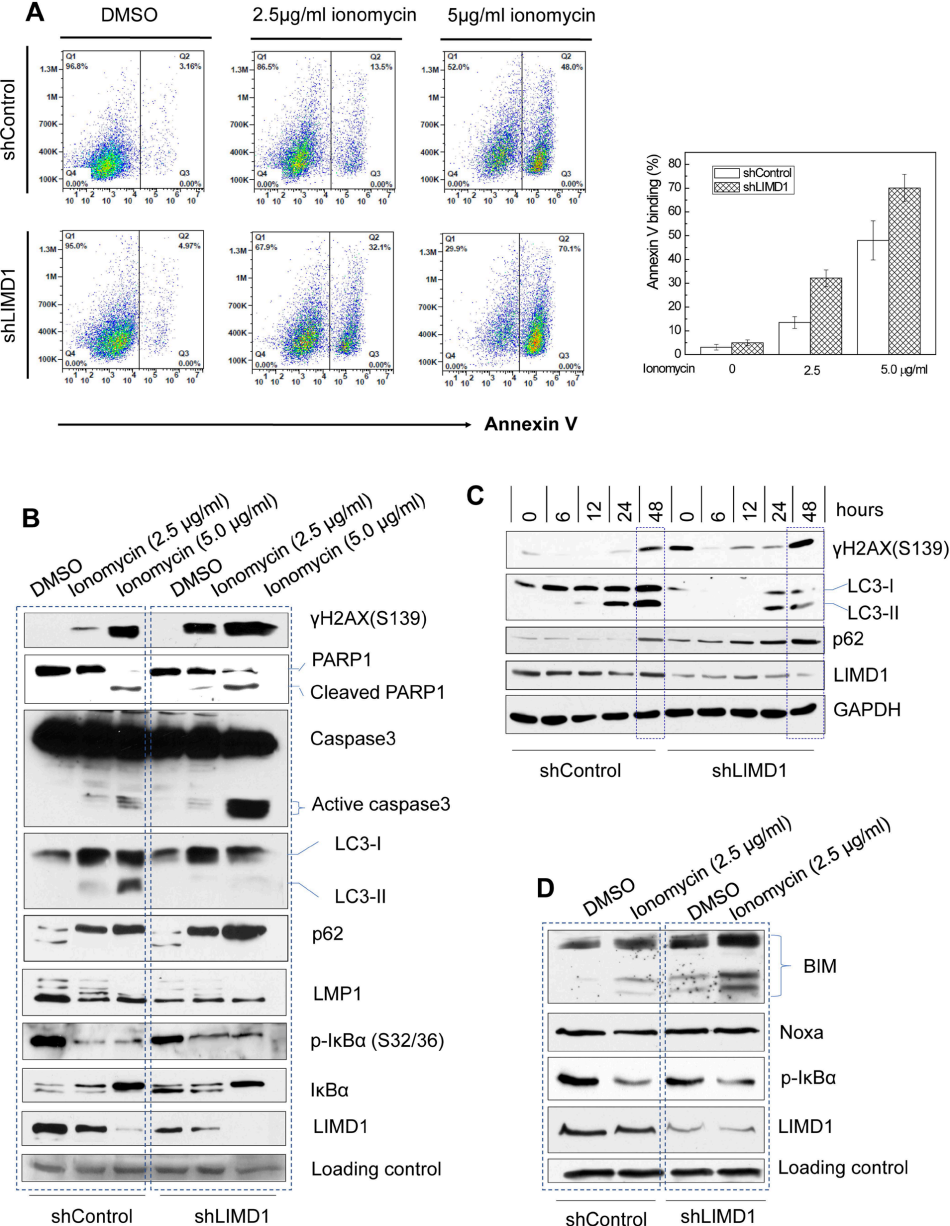
LIMD1 depletion potentiates ionomycin-induced DNA damage and apoptosis, and impairs autophagy (**A**) IB4 cell lines stably expressing LIMD1 shRNA #1 and control were treated with ionomycin for 48 h, and apoptosis was analyzed by flow cytometry for Annexin V binding. The right graph shows an analysis for a representative experiment with duplicate for each sample (mean ± SE). (**B–D**) IB4 cell lines (B) and (D) and JiJoye cell lines (C) stably expressing LIMD1 shRNA #1 and control were treated with ionomycin for 48 h (B) and (D) or time points (C). 2.5 µg/ml and 5.0 µg/ml in (B) and 2.5 mg/ml in (C) and (D). DNA damage, apoptosis, and autophagy were evaluated by immunoblotting for related hallmarks: the DNA damage hallmark γH2AX, the autophagy hallmark LC3-II, and the apoptosis hallmark Caspase 3 activity. Bim and Noxa were also analyzed.

To further assess the functional role of LIMD1 in EBV latency, we evaluated DNA damage and cell death of IB4 cells stably expressing LIMD1 shRNAs in response to ionomycin treatment. Ionomycin is an ionophore used in research to raise the intracellular level of calcium (Ca^2+^); intracellular levels of calcium influx are essential for ROS production [40], causing DNA damage. Our data show that ionomycin treatment induces apoptosis in EBV-transformed IB4 cells, as shown by Annexin V expression (Figure 6A) and caspase 3 activity (Figure 6B), and also strikingly induces DNA damage, as evidenced by expression of γH2AX, a hallmark of DNA double-strand breaks (Figure 6B). Notably, depletion of LIMD1 significantly potentiates ionomycin-induced apoptosis (Figure 6A and 6B), and remarkably increases DNA damage induced by ionomycin (Figure 6B). Taken together, our results demonstrate that LIMD1 depletion results in an enhancement in ionomycin-induced apoptosis and DNA damage.

Consistent with a previous report [41], our results show that ionomycin treatment elevates the protein level of p62, which is well known as a selective autophagy adaptor, and consequently, induces autophagy, as shown by the expression of LC3-II, a hallmark of autophagy (Figure 6B). LC3β has two forms, the cytoplasolic form LC3-I and the autophagosome membrane-bound form LC3-II; the latter is a hallmark of autophagy. However, LC3-II was not detected in cells with LIMD1 depletion. p62 itself is a target of and is degraded by selective autophagy [42]. Correspondingly, p62 is increased at the protein levels in cells with LIMD1 depletion as a consequence of impaired autophagy (Figure 6B). Our results thus indicate that LIMD1 depletion results in attenuation of ionomycin-induced, p62-mediated selective autophagy.

In contrast to p62, LMP1 protein levels are remarkably decreased in cells with autophagy, consistent with a previous report that LMP1 is degraded by autophagy [43]. Correspondingly, the anti-apoptotic NFκB activity, which is stimulated by LMP1, is also impaired by ionomycin, in line with its ability to induce apoptosis (Figure 6B). The LIMD1 protein level is decreased as well, suggesting that LIMD1 is also degraded by autophagy, or this decrease is due to transcriptional suppression by dampened LMP1/NFκB signaling (Figure 6B). Additionally, after LIMD1 depletion, LMP1 protein levels are generally lower even in the absence of autophagy (Figure 6B). This could be explained by our above results that LIMD1 depletion blocks LMP1 signal transduction, and consequently impairs NFκB activity that is involved in LMP1 autoregulation [44]. We obtained similar results from JiJoye cells that were derived from a BL cancer patient, i.e. LIMD1 depletion results in severer DNA damage, diminished autophagy and increased p62 protein levels (Figure 6C).

In general, autophagy precedes apoptosis, which occurs when the protective ability of autophagy is overcome by a stimulus such as ionizing radiation or chemotherapeutic anticancer agents; in turn, apoptosis inhibits autophagy [45–47]. The Bcl2 family plays a key role in linking these two processes [48, 49]. To explore the mechanism underneath ionomycin induction of p62-mediated autophagy and the role of LIMD1 in this process, we evaluated the expression regulation of selected Bcl2 family members, including Bim, Noxa, BNIP3, and Bcl-xL, which have been documented in autophagy [48, 49]. The protein level of the pro-apoptotic Bim, but not Noxa, is increased after LIMD1 depletion and futher elevated by ionomycin treatment (Figure 6D). Bim can inhibit autophagy by directly interacting with Beclin 1 [50]. Other tested Bcl2 family members did not change at protein levels after LIMD1 depletion (data not shown). These results suggest that LIMD1 depletion inhibits p62-mediated autophagy through upregulating Bim expression.

Taken together, our results indicate that LIMD1 confers EBV-transformed cells resistance to DNA damage and apoptosis, but renders them susceptible to autophagy, at minimum through suppressing Bim expression.

## DISCUSSION

**Figure 7:**
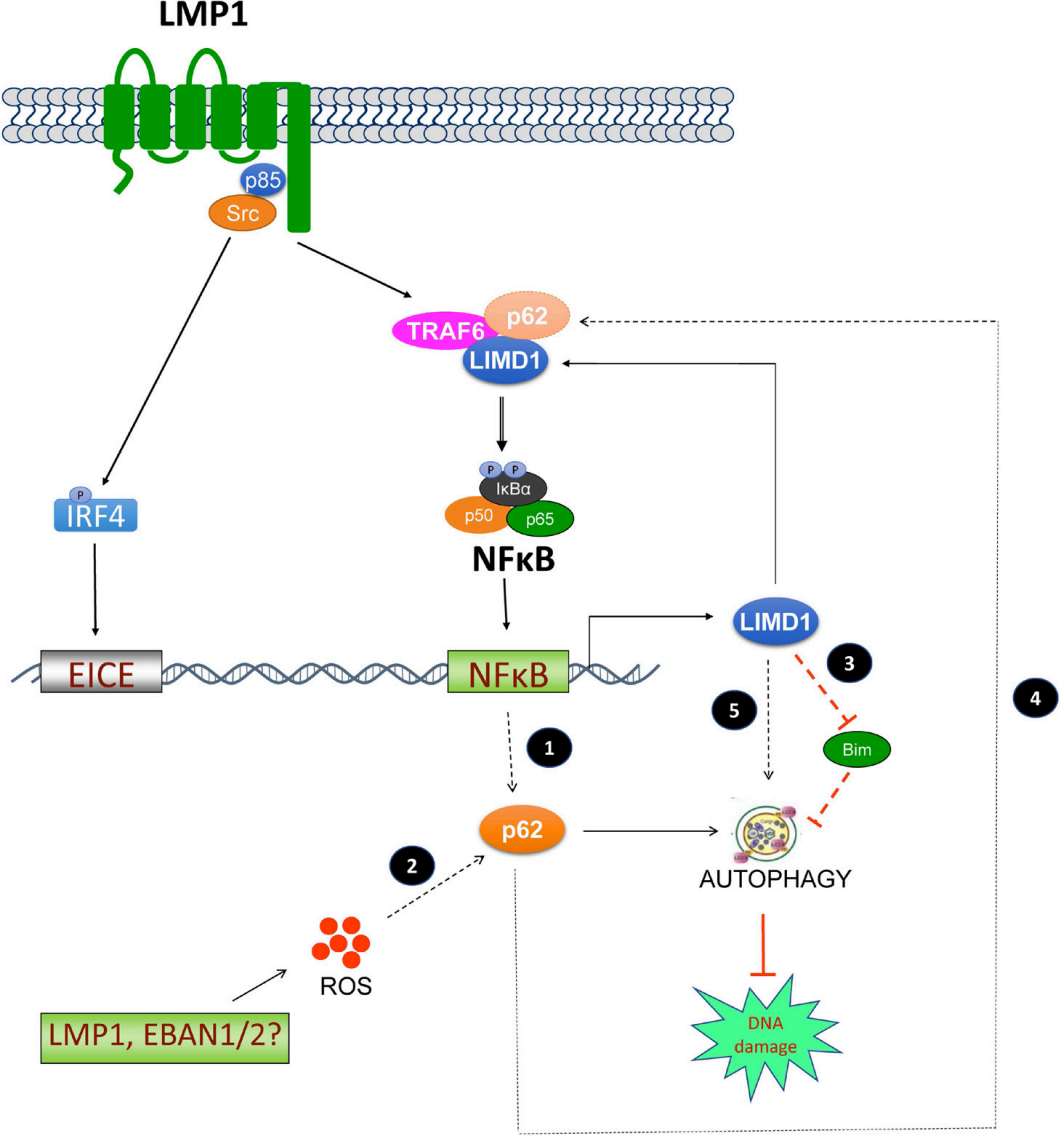
A diagram showing the interplay between LIMD1 and LMP1 LMP1 induces LIMD1 expression via NFκB and IRF4 axes. In turn, LIMD1 participates in LMP1 signal transduction by interacting with TRAF6. LIMD1 protects EBV-transformed cells from DNA damage through inducing p62-mediated autophagy that plays a crucial role in DNA repair in cancers, and this function may or may not depend on LMP1 signaling in that LIMD1 may be regulated and promotes DNA repair through other LMP1-independent mechanisms. LMP1 may induce autophagy through distinct but not fully understood mechanisms, one of which involves p62 that is likely induced by LMP1/NFκB. The pathways with broken lines represent several possible LMP1-dependent and -independent mechanisms underlying EBV regulation of p62-mediated autophagy, and are under our investigation. ① LIMD1 participates in LMP1 signal transduction to NFκB activation and ROS production, both of which induce p62; ② Other EBV factors may indirectly induce p62 expression; for example, EBNA1 and EBNA2 produce ROS that is able to induce p62; ③ LIMD1 inhibits expression of Bim that is known to inhibit p62-mediated autophagy; ④ p62 interacts with LIMD1 and TRAF6 in a multi-protein complex that facilitates NFκB activation in diverse contexts, and this mechanism may also function in LMP1 activation of NFκB; ⑤ LIMD1 may regualte p62-mediated autophagy through other LMP1-independent strategies.

In this study, we provide solid evidence for the regulation of LIMD1 expression by LMP1 through the NFκB and IRF4 signaling axes. We further show that LIMD1 is required for LMP1 signal transduction and functions. Moreover, our results have identified LIMD1 as a novel player in EBV latency and oncogenesis by protecting EBV-transformed cells from DNA damage and apoptosis but rendering them susceptible to autophagy (Figure 7).

Our results show that, in P3HR1 cells, which lack EBNA2 and LMP1, LIMD1 protein and mRNA are still expressed at considerably high levels, implying that factors other than EBNA2 and LMP1 in EBV latency contribute to LIMD1 upregulation. In fact, in addition to EBNA2, EBNA3s are also Pu.1-binding proteins, and they may induce LIMD1 expression in cooperation with Pu.1, which transactivates the LIMD1 promoter [29]. It is also notable that, when LMP1 is overexpressed in EBV-negative B cells, it caused downregulation, in lieu of upregulation, of LIMD1 expression (data not shown), and also downregulation of Bcl2 that is known to be upregulated by LMP1 in EBV latency. This parodox can be explained by the fact that LMP1 plays dual roles in cell fate; high levels of LMP1 induces apoptosis, rather than cell transformation [51, 52].

It has been reported that EBNA3A and EBNA3C cooperate with IRF4 transcriptional complex to inhibit Bim expression, and therefore protect EBV-positive cells from DNA damage-induced apoptosis [53, 54]. We show here that LIMD1 depletion results in upregulation of Bim, consistent with our conclusion that LIMD1 is required for LMP1 signal transduction in that LMP1 induces and activates IRF4 [55]. It has also been shown that, however, ionomycin-induced apoptosis is dependent of neither p53 nor Bim in EBV-positive cells [56], but depends on another pro-apoptotic Bcl2 family member Noxa [53], whose expression was not changed in response to ionomycin treatment in our experiments. Nevertheless, it is worthy of further investigation to assess the interaction between Bim and LIMD1 in EBNA3 inhibition of apoptosis in response to different chemotheraputic drugs.

LIMD1 depletion is associated with more severe DNA damage in EBV-transformed cells in response to ionomycin treatment. This consequence may be attributed by two mechanisms. The first is that LIMD1 depletion results in more DNA damage directly. The second is that LIMD1 depletion impairs a functional DNA repair machinery. Our results show that, in LIMD1-deficient cells, p62-mediated selective autophagy in response to ionomycin treatment is remarkably dampened. Since p62-mediated selective autophagy plays a crucial role in DNA repair in cancer cells [22, 23, 27], we believe that LIMD1 protects EBV-transformed cells from DNA damage by playing an indispensable role in induction of p62-mediated selective autophagy, which serves as a survival mechanism by participating in DNA repair in cancer cells that are usually deficient in traditional DNA repair mechanisms such as non-homologous end jointing (NHEJ) [14, 23].

We are now investigating potential LMP1-dependent and independent mechanisms underlying the interplay between p62-mediated autophagy and DDR in EBV latency. In fact, we show here that LIMD1 depletion promotes Bim protein expression, and Bim is known to inhibit autophagy [50]. As a second possible mechanism, LIMD1 is required for LMP1 signaling, which is known to regulate unfolded protein responses and autophagy through multiple mechanisms that have not been fully disclosed, including its ability to induce autophagy through its N-terminal six transmembrane domains and its ability to induce ROS that induces autophagy [17, 43, 57–61]. Third, p62 is known to be upregulated in response to ROS production that is induced individually by three LMP1 latent proteins, including LMP1, EBNA1, and EBNA2 [36, 57, 62]. Fourth, it is interesting that p62 interacts with LIMD1 in the multi-protein complex LIMD1-p62-TRAF6-PKCζ that regulates IL1 and RANKL signaling [7, 32]. The association between p62 and TRAF6 also facilitates NFκB activation in Ras, TNFR, nerve growth factor (NGF), and Toll-like receptor (TLR) signaling pathways [63–67]. Considering that LMP1 interacts with p62 in a high throughput screen [68], it is conceivable that both LIMD1 and p62 interact with TRAF6 downstream of LMP1 signaling and cooperate to regulate the interplay between LMP1-mediated DDR and autophagy.

LIMD1 is deemed a tumor suppressor [6]. It represses the anti-oncogenic Hippo pathway by antagonizing YAP1 phosphorylation [11, 69], and promotes ubiquitination-mediated HIF1α degradation by interacting with the tumor suppressor VHL [9, 10]. It is also downregulated, and inhibits NFκB activity and autophagic cell death, in human non-small cell lung cancer cells [12]. Surprisingly, its expression is positively correlated with expression of the oncogene IRF4 in EBV latency, and it is required for LMP1 oncogenic functions and its depletion promotes DNA damage and apoptosis. It is also overexpressed in and is a hallmark of ABC DLBCL [5]. Further, our results indicate that LIMD1 is downregulated at transcriptional and/or post-translational levels, in cells either with LMP1 overexpression that induces cell death or in response to ionomycin treatment that induces DNA damage and apoptosis. Thus, we believe that LIMD1 plays an oncogenic role in EBV-associated lymphomas and other hematological malignancies, although further verification in animal models is required. The paradox roles of LIMD1 may depend on its post-translational modifications; cell-cycle-dependent phosphorylation may play a role in its function as a tumor suppressor [70].

Our intriguing observations open a novel research avenue in the field of EBV oncogenesis that involves DDR, apoptosis, and autophagy, and their interplay mediated by LIMD1 and p62 in EBV oncogenesis, which have never been reported. We believe that future in-depth mechanistic studies will provide key innovative insights into EBV oncogenesis.

## MATERIALS AND METHODS

### Constructs, antibodies, and reagents

pGL4.10/LIMD1p(-1990/+50)-Luc2 and its mutant with the Pu.1-binding site mutated were described previously [29]. 3XFlag-tagged LMP1 and mutants, and other expression constructs were described in our recent paper [55]. Deletion and point mutants were generated by subcloning or site-directed mutation (Stratagene), and verified by sequencing. LIMD1 cDNA was amplified from IB4 cell line with the primer pair: forward: 5′-CCGGAATTCATGGATAAGTATGACGACCTGG-3′ and reverse: 5′-GCTCTAGACTAGAAGTGGTGCTGGTGAAGG-3′, and cloned into pcDNA3 at EcoRI and XbaI sites and verified by sequencing. The set of LIMD1 shRNAs that includes 6 LIMD1 shRNAs constructed in the lentiviral vector pTRIPz was purchased from Open Biosystems. We chose two of them with the highest knockdown efficiencies for loss-of-function assays.

LMP1 mouse monoclonal antibody (clone CS1-4) was purchased from Dako. IRF4 mouse monoclonal antibody (clone MUM1p) and goat polyclonal antibody (clone M17), LIMD1 mouse monoclonal antibody (clone H4), and p62 mouse monoclonal antibody (D-3) were from Santa Cruz for immunoprecipitation and immunoblotting. LC3b rabbit polyclonal antibody was from Invitrogen. γH2AX(S139) monoclonal antibody was from BioLegend. TRAF6 rabbit monoclonal antibody (clone 1H4L2) and rabbit polyclonal antibody (clone H274) were from ABfinity and Santa Cruz, respectively. p-p38(Thr180/Tyr182), p-IκBα (s32/36), p-PTEN(S380), and Bim (C34C5) antibodies were purchased from Cell Signaling Technology. Flag (clone M2) antibody was from Sigma. Goat anti-mouse IgG-HRP, mouse anti-rabbit IgG-HRP, and mouse anti-goat IgG-HRP, and all other primary antibodies were purchased from Santa Cruz. BAY11-7085, ionomycin calcium salt, and doxycycline (Dox) were purchased from Sigma.

### Cell lines

293 and 293T are human kidney epithelial cell lines. SavI, SavIII, P3HR1 and JiJoye are human B cell lines derived from EBV-positive Burkitt’s lymphoma patients. P3HR1 was derived from JiJoye but does not express LMP1 due to lacking the entire EBNA2 ORF in the viral genome [71]. The LCL line IB4 was derived from umbilical cord B-lymphocytes latently infected with EBV *in vitro*. CEM is a HTLV1-negative, EBV-negative T cell line derived from acute leukemia, and MT4 is a HTLV1-transformed CD4^+^ T cell line derived from umbilical cord blood lymphocytes. Epithelial cells are cultured with DMEM plus 10% FBS and antibiotics, and B and T cells are cultured with RPMI1640 medium plus 10% FBS and antibiotics. All cell culture supplies were purchased from Life Technologies.

### Transfection

Lentiviral packing, preparation, infection, and selection of stable cells by puromycin were performed as detailed in our previous publication [31, 55]. LIMD1 shRNA expression was induced by 1 µg/ml DOX. For other transfection of B cells, the Nucleofector kit for human B cells (Lonza) or the Gene Pulser Xcell system (Bio-Rad) was used. 293 and 293T cells were transfected with Effectene (Qiagen) or Fugene HD (Promega).

### Promoter-reporter assays

293 cells were transfected with expression plasmids as indicated together with LIMD1p-Luc2 (or its mutants) and Renilla as internal transfection control. Empty vector was used to equalize the total amounts of DNA in all transfections. Cells were collected 24 h after transfection. Luciferase activity was measured with equal amounts (10% of total for each sample) of protein lysates with the use of a Dual Luciferase Assay kit (Promega), on a multimode microplate reader (Turner Biosystems). Results are the mean ± standard error (SE) of duplicates for each sample. At least three consistent results were obtained from independent experiments and representative results are shown. The ability of the empty vector controls to activate the promoter constructs was set to 1.

### Immunoprecipitation and immunoblotting

For endogenous protein interaction, 1 × 10^7^ cells were used for each IP. For interaction between transiently expressed proteins, 293T cells in 60-mm dishes were collected 48 h after transfection. Cells were lysed with NP40 lysis buffer (150 mM NaCl, 1% NP-40, 50 mM Tris-pH 8.0, plus protease inhibitors), and cell lysates were subjected to immunoprecipitation with 1.5 µg indicated antibodies for overnight, and then incubated with 40 µl Protein A/G beads (Santa Cruz) for 1 h. After three washes, proteins on beads were denatured before separated by SDS-PAGE. Immunoblotting was carried out with indicated antibodies and signals were detected with an enhanced chemiluminescence (ECL) kit following the manufacturer’s protocol (Amersham Pharmacia Biotech).

### Real-time quantitative PCR

Quantitative PCR (qPCR) was performed with the use of SYBR Green (Applied Biosystems), on a CFX96™ Real-time PCR Detection System (Bio-Rad Laboratories, Inc.). All reactions were run in duplicates. Mean cycle threshold (*C*_t_) values were normalized to 18 s rRNA, yielding a normalized C_*t*_ (Δ*C*_t_). ΔΔ*C*_t_ value was calculated by subtracting respective control from the Δ*C*_t_, and expression level was then calculated by 2 raised to the power of respective –ΔΔ*C*_t_ value. The averages of 2^(–ΔΔ*C*_t_) in the control samples were set to 1 or 100%. Results are the average ± standard error (SE) of triplicates for each sample. Primers for real-time qPCR are as follows: LIMD1: F: 5′-TGGGGAACCTCTACCATGAC-3′ and R: 5′-CACAAAACACTTTGCCGTTG-3′; p62: F: 5'-TG CTAGGCCAGTGAAGGGAG-3' and R: 5'-CTTGTCTG TTGTGGGTAAAGCAAC-3'; IRF4: F: 5′-CGGGCAA GCAGGACTACAAC-3′ and R: 5′-CCTTTAAACAGT GCCCAAGCC-3′; Bcl6: F: 5′-CGCAACTCTGAAGA GCCACCTGCG-3′ and R: 5′-TTTGTGACGGAAATG CAGGTTA-3′. 18 s rRNA: F: 5′-GGCCCTGTAATTG GAATGAGTC-3′ and R: 5′-CCAAGATCCAACTACGA GCTT-3′.

### Apoptosis assay

Apoptosis was assayed using flow cytometry as detailed in our previous publication [29], for Annex V binding (BD Biosciences, San Jose, CA). Caspase 3 activity and apoptosis-related proteins including Bim expression were evaluated by Western blotting.

### Chromosome immunoprecipitation (ChIP)

ChIP was performed in 293 cells as described in our previous publication [55], with the use of ChIP-IT Express Enzymatic kit (Active Motif). qPCR was performed with the human LIMD1 promoter EICE primers: 5′- AA GGCTGCGGCAAGGGGCCG-3′ (forward) and 5′-CACC AGGCCTGACTCCTTGG-3′ (reverse), and the NFκB- binding site primers: 5′-TGCGCGCAGGCACAACG AG-3′ (forward) and 5′- CGTGTCACCCATGGCTGG-3′ (reverse).

### Statistical analysis

Unpaired, two-tailed student *t* tests were executed using Graphpad Prism (version 5) to determine the differences between two data sets obtained from three independent experiments. *p <* 0.05 (^*^) and *p <* 0.01 (^**^) were considered significant and *p <* 0.001 (^***^) was considered very significant. Data are expressed as mean ± standard error (SE) of duplicate or triplicate samples, and representative results from at least three with similar results are shown.
